# Long distance electron transfer through the aqueous solution between redox partner proteins

**DOI:** 10.1038/s41467-018-07499-x

**Published:** 2018-12-04

**Authors:** Anna Lagunas, Alejandra Guerra-Castellano, Alba Nin-Hill, Irene Díaz-Moreno, Miguel A. De la Rosa, Josep Samitier, Carme Rovira, Pau Gorostiza

**Affiliations:** 1 Institute for Bioengineering of Catalonia (IBEC), The Barcelona Institute of Science and Technology, Barcelona, 08028 Spain; 2 Networking Biomedical Research Center (CIBER), Madrid, 28029 Spain; 3 Institute of Chemical Research (IIQ), Centre of Scientific Research Isla de la Cartuja (cicCartuja), University of Sevilla-CSIC, Sevilla, 41092 Spain; 4 Inorganic and Organic Chemistry Department & Institute of Theoretical and Computational Chemistry (IQTCUB), University of Barcelona (UB), Barcelona, 08028 Spain; 5 Department of Electronics and Biomedical Engineering, University of Barcelona (UB), Barcelona, 08028 Spain; 6 Catalan Institution for Research and Advanced Studies (ICREA), Barcelona, 08010 Spain

## Abstract

Despite the importance of electron transfer between redox proteins in photosynthesis and respiration, the inter-protein electron transfer rate between redox partner proteins has never been measured as a function of their separation in aqueous solution. Here, we use electrochemical tunneling spectroscopy to show that the current between two protein partners decays along more than 10 nm in the solution. Molecular dynamics simulations reveal a reduced ionic density and extended electric field in the volume confined between the proteins. The distance-decay factor and the calculated local barrier for electron transfer are regulated by the electrochemical potential applied to the proteins. Redox partners could use electrochemically gated, long distance electron transfer through the solution in order to conciliate high specificity with weak binding, thus keeping high turnover rates in the crowded environment of cells.

## Abstract (web summary)

Electron transport chains rely on interactions between redox proteins, but the distance-dependence of the electron transfer rate through the solution is unknown. Here, the authors show that the current between two redox protein partners occurs at long distances and is electrochemically gated.

## Introduction

Electrons flow through biological membranes, yet they are not transported by passive conductive wire structures. Instead, they are carried individually by redox proteins, whose diversity of interactions and electrochemical properties can meet multiple biochemical functions and regulation requirements^1,2^. As a consequence, the demands on the electron transfer (ET) capabilities of these proteins are conflicting: their binding must be tight in order to keep ET rates high, but binding should be sufficiently weak to allow a high turnover rate and overall ET efficiency. These demands can be traded off by a stepwise association process between the protein partners: as the distance between redox partners is reduced, an initial encounter complex is formed that leads to a final active complex in which ET occurs between the redox-active sites located within nanometer-scale proximity^3^. The structure of some active complexes between redox protein partners has been revealed by X-ray crystallography^4^. However, substantial differences can be observed in the distance between the active sites of several redox protein partner complexes, which raises the question of whether ET between proteins already occurs while the protein is approaching its partner site^4^. The distance-dependence of interprotein ET has been studied in various protein partners and mutants that alter the geometry of the wild-type complex, which leads to different distances between active sites, and coupling mechanisms^5^. However, these studies portrait the final bound state(s) of the partner proteins, rather than the dynamic events leading to complex formation^2^.

ET studies in proteins have mostly focused on intramolecular transfer because of its relative simplicity. In this case, ET has been shown to proceed by tunneling with a distance decay factor (*β*) of 11 nm^−1^, and is limited to a distance between donor and acceptor of 2.5 nm if a single step is involved^6^. Interprotein ET between diffusing proteins in solution is more complex to interpret than intramolecular ET because of the wide range of geometries that the partners may access^1^. The *β* value of ET between a protein active site and a metallic electrode has been measured by changing the thickness of molecular monolayers on a metal surface^7^, and more directly by varying the aqueous gap separation between the protein and the metallic probe of an electrochemical scanning tunneling microscope (ECSTM)^8^.

Here, we aim to obtain, for a single redox protein pair, a functional readout of the ET rate during the steps involved in the transition from the uncoupled partner proteins to binding, complex formation, and ET. Specifically, the objective is using electrochemical tunneling spectroscopy (ECTS)^9,10^ under bipotentiostatic control to measure the current between two ET partner proteins and its dependence with their separation in an aqueous electrolyte.

We observe that current decays along more than 10 nm in the solution, corresponding to distance decay factors that are tenfold lower than those reported for protein-mediated tunneling or for tunneling through organic or water glasses. Molecular dynamics simulations reveal a reduced ionic density and extended electric field in the volume confined between the proteins. In addition, the distance decay factor is regulated by the electrochemical potential applied to the proteins, which highlights the physiological relevance of these results.

## Results

### Long-distance electron transfer between protein partners

We chose the ET process between cytochrome *c* (C*c*) and the mitochondrial complex III (CIII), or cytochrome *bc*_1,_ as a well-known and representative step of the mitochondrial respiratory chain^11,12^. C*c* forms a natural complex with the cytochrome *c*_1_ (C*c*_1_) subunit of cytochrome *bc*_1_. Although human C*c*_1_ has not yet been obtained in soluble form, this interaction can be conveniently studied within the well-characterized cross complex between human cytochrome *c* (hC*c*) and the soluble domain of plant cytochrome C*c*_1_ (pC*c*_1_)^13^, which were respectively bound to the ECTS probe and sample electrodes. In particular, the E104C hC*c* mutant (hereinafter hC*c*) was chosen because this cysteine is located opposite to the redox-active heme group. In this way, upon covalent binding to the probe electrode, the orientation of the hC*c* active site is directed toward the solution, and the rotational freedom of the thiol bond facilitates the interaction with its partner. In addition, E104C hC*c* displays high conductivity in films^14^. The attachment of the soluble domain of pC*c*_1_ by means of its N-terminal Cys10, also exposes its active site to the solution and provides rotational freedom. Therefore, the hydrophobic pockets surrounding the heme groups of hC*c* and pC*c*_1_ can face each other and interact during the experiments (Supplementary Figure 1).

**Fig. 1:**
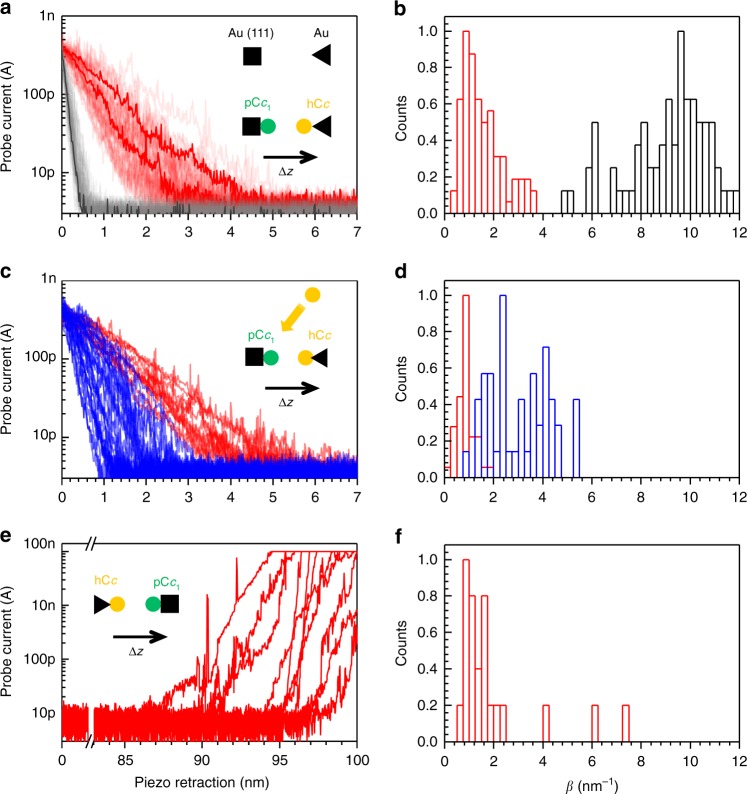
Current–distance electrochemical tunneling spectroscopy of pC*c*_1_–hC*c*. **a** Ensemble of semilogarithmic current–distance (I-z) plots obtained for pC*c*_1_–hC*c* proteins (red) and for bare gold (gray) during probe retraction, showing a more gradual current decay for pC*c*_1_–hC*c*. Selected representative traces are depicted in bold. Sample and probe electrodes are represented by a square and a triangle, respectively. **b** Histograms of distance decay factors (*β*) quantified from individual curves in **a**. **c** Inhibition experiment conducted to test the specificity of pC*c*_1_–hC*c*. Semilogarithmic I-z plots obtained for pC*c*_1_–hC*c* during probe retraction before (red), and after (blue) the addition of WT hC*c*. The measured probe current is reduced more abruptly upon WT hC*c* addition. **d** Histograms of *β* from individual curves in **c**. **e** Selected semilogarithmic I-z plots obtained for pC*c*_1_–hC*c* obtained in approach experiments after retracting the probe 100 nm. **f** Histogram of *β* obtained from individual curves in **e**. All the experiments were performed at *U*_s_ = −200 mV and at constant bias of 800 mV in 50 mM sodium phosphate buffer, pH = 6.5. Initial current set point 0.4 nA

ECTS experiments were initially performed at electrochemical potentials, in which pC*c*_1_ on the sample electrode is reduced (U_S_ = −200 mV) and hC*c* on the ECTS probe is oxidized (*U*_P_ = 600 mV), resulting in a constant positive bias *U*_bias_ = *U*_P_–*U*_S_ = 800 mV. Under these conditions, electrons are transferred from pC*c*_1_ to hC*c* in analogy to physiological ET (Supplementary Figure 1). To obtain each I-z plot, the current between the probe and sample was initially set to 0.4 nA. Upon disconnection of the feedback loop, the piezoelectric scanner holding the probe was retracted at 12 nm/s while the current was recorded. The feedback was then restored and the probe reapproached, allowing subsequent recordings at different locations on the sample. I-z plots obtained with bare gold sample and probe electrodes show that the current is exponentially reduced below 10 pA in less than 1 nm (gray plots in Fig. 1a; note that a distance-independent faradaic leakage current is measured below a few pA depending on the probe insulation). The corresponding distance decay factors are distributed around *β* = 9 ± 2 nm^−1^ (black histogram in Fig. 1b), in agreement with previous studies^10^. Strikingly, I-z plots for sample-bound pC*c*_1_ and probe-bound hC*c* span several nanometers (red plots in Fig. 1a) with a nearly exponential dependence that allows inferring a distance decay factor, which is distributed around 1.5 ± 0.8 nm^−1^ (red histogram in Fig. 1b).

These *β* values are directly measured from the current at constant electrochemical potential, during retraction of an accurately calibrated piezoelectric positioner. Thus, they are not subject to models or approximations. In order to rule out protein-related artifacts, we verified that such long-distance currents are specific between pC*c*_1_ and hC*c*. First, we added wild-type human C*c* (WT hC*c*) without C-terminal cysteine to the medium at a concentration that saturates the pC*c*_1_ immobilized on the gold sample, in order to inhibit the pC*c*_1_–hC*c* interaction. The decay of I-z curves between pC*c*_1_ and hC*c* (red plots in Fig. 1c) became more pronounced upon addition of WT hC*c* (blue plots in Fig. 1c), doubling the *β* value to 3 ± 1 nm^−1^ and broadening the distribution (blue histogram in Fig. 1d). Similarly, the *β* value of the non-specific interaction between hC*c*-functionalized probe and sample (self-ET) nearly doubled the *β* of the specific pC*c*_1_–hC*c* interaction (Supplementary Figure 2). The interaction between each protein and a bare gold electrode produced even higher *β* values (Supplementary Figure 3). Finally, since subsequent I-z retraction curves might be subject to post-interaction effects, such as direct protein contact and protein deformation upon probe retraction, we also recorded I-z curves by approaching the electrodes. Prior to these measurements, the probe was retracted 100 nm from the sample surface (initial set point at 0.4 nA), which is a distance longer than the extended protein length. Although the risk of probe crashing in these I-z recordings was higher, the current increase was again nearly exponential (Fig. 1e) with a distribution of *β* values similar to that obtained in retraction curves (Fig. 1f). Thus, the observations of Fig. 1 (red plots) correspond to interprotein ET. Note that the distance-dependence of the measured current is assumed to arise mainly from ET between pC*c*_1_ and hC*c*, but electron transport between the probe and substrate also involves ET between probe–hC*c* and substrate–pC*c*_1_.

Such a gradual current decay is not compatible with tunneling at room temperature, and ET is more likely supported in this case by hopping or some process other than electron tunneling^6^. ET spanning lengths up to 7 nm (corresponding to *β* values below 1 nm^−1^) have been observed in peptides^15^, in ferritin complexes^16^, and in polymers with redox groups^17,18^, which are believed to act as “bridge” mediators of superexchange tunneling and hopping. In the absence of such redox mediators in the aqueous space between the proteins, the low decay factor (Fig. 1) cannot be accounted for in calculations of pathways including only through-bond and through-space segments, as it is usually assumed from crystal structures of protein complexes^5,6^.

### Role of the aqueous solution in long-distance electron transfer

To account for these results, we hypothesized an ET pathway through the solution where water molecules are tunneling mediators in the constrained aqueous interface between protein partners. In this local region, the dipole resonance of oriented water layers leads to multiple intermediate states or “channels” through which electrons can switch^9^. Similar mechanisms were observed in simulations of ET between metallic electrodes^19^ and of cytochrome b_5_ self-ET^20^. Experimentally, ET enhancement has been linked to a lower energy barrier caused by discrete water layers in the gap between metallic electrodes^9^, and was also reported between a semiconductor and multiheme C*c*^21^. In the latter study, ET distances increased upon decreasing ionic strength, leading to *β* = 5 nm^−1^ at 1 mM, and we observe a similar trend with even lower *β* values in pC*c*_1_–hC*c* (red plot in Supplementary Figure 4). The influence of the ionic concentration on I-z profiles between the proteins suggests that the conduction process depends on the electrical double layer at the surface. In a low ionic concentration solution, the electric potential extends along several nanometers near the surface of an electronic conductor, within the diffuse ionic layer (Gouy–Chapman layer)^22^. Thus, the situation could be similar in the aqueous gap between proteins due to ion depletion near the active sites.

**Fig. 2:**
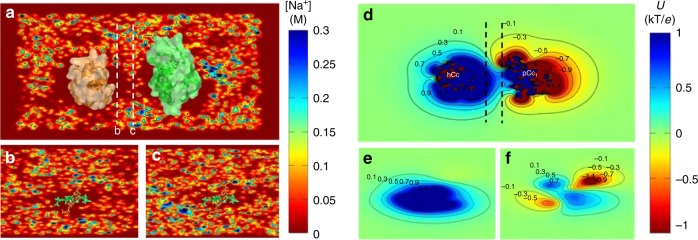
Ion concentration and electrostatic potential around hC*c* and pC*c*_1_ from MD simulations. **a** Side view of the averaged sodium concentration (M) map, which is relatively low in the region between the proteins, thereby reducing ionic charge screening. hC*c* (orange) and pC*c*_1_ (green) proteins are superimposed for visualization purposes. Each contour line corresponds to 0.15 M. **b**, **c** Example cross sections corresponding to the dashed white lines in **a**. The two planes are separated 0.6 nm. The corresponding heme groups (hC*c* protein, orange, and pC*c*_1_ protein, green) are also placed on the figure for visualization purposes. **d** Side view of the equipotential lines from −1.1 kT/*e* (red) to 1.1 kT/*e* (blue) calculated by Poisson–Boltzmann equation (APBS method); ion concentration is 50 mM, pH = 6.5. Each contour line corresponds to 0.2 kT/*e*. **e**, **f** Cross sections corresponding to the dashed white lines in **d**, the two planes are separated 0.6 nm

In order to study the distribution of ions at the pC*c*_1_–hC*c* interface, we performed molecular dynamics (MD) simulations of pC*c*_1_ and hC*c*, whose redox-active sites were facing each other at 3 nm separation, in a solvent with explicit water molecules and 50 mM ionic concentration (see Methods section for details). In this configuration, anions bind to basic residues in the charged ring of lysines of hC*c* (K13, K27, K72, K73, K79, K86, and K87) but are otherwise homogeneously distributed between the active sites of the proteins (indicated by the heme groups in Supplementary Figure 5). However, the cationic concentration (Fig. 2a) is significantly reduced at the interface and nearly zero in the proximity of the hC*c* active site (Fig. 2b–d). The computed electrostatic potential within a 50 mM ionic concentration shows equipotential lines that are extended between the two proteins forming a conduit (Fig. 2d–f).

The concentration of water molecules is homogeneous between the proteins except near the surface of hydrophilic residues (e.g., Q16 and K13 of hC*c* in Supplementary Figure 6) where it is significantly higher. Bound water molecules are also observed in the crystal structure of many redox protein active sites and act as mediators of their ET pathways^23–25^. The importance of water dynamics around key residues in these regions has also been highlighted in MD simulation studies^20,26^.

In view of the simulation results, we asked if low ionic concentrations are sufficient to observe long-distance ET between the electrodes, regardless of the presence of the proteins. I-z curves obtained in bare gold at 50 mM phosphate buffer (Fig. 1a and Fig. 1b) show that long-distance ET is not sustained at this buffer concentration. However, I-z measurements of bare gold in pure water (Supplementary Figure 7a) yield decay factors similar to those obtained for pC*c*_1_–hC*c* (Fig. 1a), yet more broadly distributed (Supplementary Figure 7b). The local barrier height (Φ_local_) can be calculated directly from I-z plots (see Supplementary Information)^27^. In bare gold at high ionic concentration (50 mM), barrier values oscillate in the range 0.5–1.2 eV with a spatial periodicity of 0.35 nm corresponding to the diameter of water molecules (Supplementary Figure 8a), as previously reported^27^. In contrast, the local barrier along the gap is reduced below 50 meV both between bare gold electrodes in pure water (Supplementary Figure 8b) and between pC*c*_1_ and hC*c* in the 50 mM solution (Supplementary Figure 9), in agreement with studies in metals^9^ and proteins^21^.

### Electrochemical gating of long-distance electron transfer

**Fig. 3:**
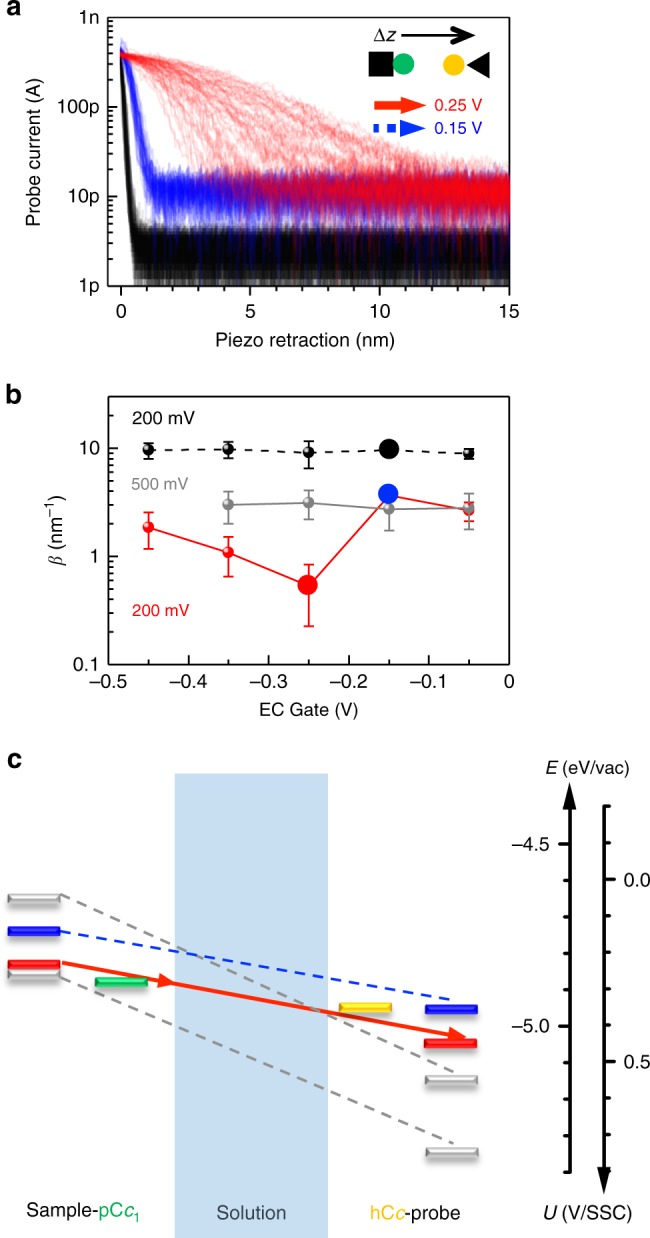
Electrochemical gating of long-distance electron transfer between pC*c*_1_ and hC*c*. **a** I-z plots at 200 mV constant bias for pC*c*_1_–hC*c* at *U*_P_ = 0.45 V and *U*_S_ = 0.25 V (red), and at *U*_P_ = 0.35 V and *U*_S_ = 0.15 V for pC*c*_1_–hC*c* (blue) and for bare gold (black). Red curves (*β* = 0.5 ± 0.3 nm^−1^) show long- distance ET beyond 10 nm. **b** Averaged *β* values (mean ± SD of *N* = 80) vs. EC gate potential at 200 mV constant bias for bare gold (black) and pC*c*_1_–hC*c* (red), and at 500 mV constant bias for pC*c*_1_–hC*c* (gray). Colored data points correspond to curves shown in **a**. **c** Energy diagram of ET processes for pC*c*_1_–hC*c* in the conditions shown in **a** and **b** (red: *U*_P_ = 0.45 V, *U*_S_ = 0.25 V; blue: *U*_P_ = 0.35 V, *U*_S_ = 0.15 V). ET processes at 500 mV constant bias are shown in gray (*U*_P_ = 0.55 V, *U*_S_ = 0.05 V; *U*_P_ = 0.75 V, *U*_S_ = 0.25 V). Fermi energies (sample, probe, and pC*c*_1_ and hC*c* redox levels) are in the absolute energy scale *E* (eV) = –e·*U* (V/SSC)−4.6 eV. The *β* is lowest when Fermi levels are closest to the redox levels of pC*c*_1_ and hC*c*

To further investigate the specificity and physiological relevance of long-distance ET between redox partner proteins, we asked if the local barrier between pC*c*_1_ and hC*c* is altered around their redox potentials at moderate bias, near biological membrane potentials. Strikingly, I-z plots at different probe and sample potentials display a marked potential dependence, with *β* and Φ_local_ reaching minima near the redox potentials of pC*c*_1_ and hC*c*, in stark contrast with the potential-independent *β* and Φ_local_ between metallic electrodes (Fig. 3 and Supplementary Figure 10). These electrochemical gating experiments^28^ were performed at different probe and sample potentials that keep a constant moderate bias of 200 mV and a higher bias of 500 mV (see the Supplementary Information for experimental details and the definition of the electrochemical gate potential, EC gate). Average *β* values obtained as a function of the EC gate display a minimum of 0.5 ± 0.3 nm^−1^ at −0.25 V EC gate potential for pC*c*_1_–hC*c* and allow long-distance ET beyond 10 nm (red plot in Fig. 3a and red dots in Fig. 3b). This potential value corresponds to U_S_ close to the midpoint redox potentials of pC*c*_1_ (0.28 V vs. SSC) and hC*c* (0.35 V vs. SSC) (Supplementary Figure 10). Thus, the minimum of *β* and the maximum spatial extent of long-distance ET occur when the Fermi levels of the electrodes and the redox levels of pC*c*_1_ and hC*c* are approximately aligned and electrons can be transferred in quasi-isoenergetic conditions (Fig. 3c). No minimum was observed for bare gold in the same potential range (black dots in Fig. 3b), which highlights the contribution of the redox centers in ET between pC*c*_1_ and hC*c*. No changes in *β* were observed in gating experiments at more positive probe potentials, far from the hC*c* redox midpoint (500 mV bias, gray dots in Fig. 3b). As a figure of merit of the different *β* values, note that 1 nm away from the 0.4 nA set point position (red plot in Fig. 3a), the current between hC*c* and pC*c*_1_ near their redox potentials is tenfold higher than the current out of resonance (blue plot in Fig. 3a). The on/off ratio of *β* in these conditions is around 7 (red and blue dots in Fig. 3b). Thus, the redox state of the proteins strongly regulates the decay rate at moderate probe–sample bias (200 mV), which shows that ET is electrochemically gated in a narrow potential and spatial range. The control of ET rate by small changes in the redox potential suggests a plausible biological role for long-distance ET through aqueous solution.

## Discussion

The atomic structure of the yeast CIII–C*c* complex^4^ revealed the “core interface” of C*c*, i.e., the minimal site that ensures a geometry suited for ET and a central feature of the interaction between C*c* and its functionally diverse redox partners. It was proposed that protein–protein recognition is mediated by the charged rings around the hydrophobic patches and that the function of the latter is to expel the solvent, leaving a layer of water molecules entrapped at the interface in the final interaction state^4^. The dynamic intermediate steps leading to the final complex formation have been difficult to address experimentally and are the subject of debate^3^. In these processes, knowledge on the molecular structure of the solution between the proteins largely relies on models and MD simulations, and new methods are required to elucidate it. On hydrophilic materials surfaces, water is densely packed, strongly oriented, and weakly hydrogen-bonded with its neighbors, whereas it presents low density, slow orientation dynamics, and bulk-like hydrogen bonding next to hydrophobic surfaces^29^. Although water structuring does not extend beyond a few layers in the vicinity of hydrated solutes and open surfaces, hydrogen-bonded water chains and networks have been observed in confined volumes of water channels, enzymes, and ET proteins^20,30^. Since water has an active role in ET at solid/liquid interfaces^27^, it must be taken into account to calculate the overall ET rate between redox sites^20,26^. However, considering that charge screening is high in physiological solutions (the Debye–Hückel length, κ^−1^, is around 1 nm at 100 mM), we wondered how the electrical driving force is maintained beyond a few water layers from the open protein surface and leads to the observed long-distance currents (Fig. 1). The MD simulations of Fig. 2 and Supplementary Figure 6 show that the ring of lysines in hC*c* accumulates anions, and that cations are depleted from the hydrophobic patch in a region that extends between the proteins. Such ionic distribution supports a reduced electrical screening, long κ^−1^, and explains that the potential profile in I-z curves extends along several nanometers (Fig. 2d), following an exponential dependence as a result of Maxwell–Boltzmann statistics (Gouy–Chapman layer)^22^. This behavior is in contrast to the abrupt potential drop of the Helmholtz layer in protein surface regions exposed to high ionic concentration (Fig. 2d).

Although the electronic and physicochemical properties of metal and protein surfaces are very different, I-z recordings between gold electrodes in pure water (Supplementary Figure 7) yield low *β* and Φ_local_ as observed in experiments with pC*c*_1_ and hC*c* in a 50 mM electrolyte (Fig. 1, Supplementary Figures 8 and 9). The fact that the spatial extent of the current and of the potential between proteins is similar (red plot in Supplementary Figure 4) indicates that the observed long-distance ET current is driven by the diffuse double layer. In order to account for our experimental observations and MD simulations, we interpret that when pC*c*_1_ and hC*c* approach, a region of reduced ionic concentration is formed in the confined volume between their hydrophobic patches. The weak electrical screening allows the potential profile *U*(*z*) to extend along several nanometers between the proteins and to give rise to an electric field all the way (*E* = d*U*/d*z*). Thus, the Gouy–Chapman region can act as a charge conduit for long-distance ET through the aqueous solution. The identity of charge carriers and the role of water in the ET reaction remain to be elucidated. One possibility is that the enhanced electronic conduction is due to ET active small molecules in the vicinity of the proteins, as farther “leaks” throughout the solution would reduce the specificity of ET and cause biological damage.

The direct experimental observation of electrochemically gated, long-distance ET between pC*c*_1_ and hC*c* through the aqueous solution is relevant to understand interprotein ET and its regulation. By molding the ionic distribution between their active sites, these redox protein partners could use long-distance ET to conciliate high specificity with weak binding, in order to keep the high turnover rate required by their biological function. Thus, a well-defined, static protein complex may be not a strict requirement for ET between them^2^, and ET could occur prior to tight binding if partners are suitably oriented and their redox potentials are matched, for example in complexes that only exist in the encounter state^3^. This notion is also in agreement with the idea that the ET complex that crystallizes is only one of a family of kinetically competent complexes^31^, and has important consequences for the overall efficacy of redox protein ET in the crowded environment of the cell. In particular, the recently reported structures of mitochondrial respiratory supercomplexes^32–34^ highlight that electrons must be shuttled along 10 nm distances by C*c* between the active sites of CIII and CIV. This process could be efficiently bridged by C*c* proteins^13^ located near the active sites in two sequential long-distance ET steps without requiring slow diffusion and binding–unbinding events.

## Methods

### Sample preparation

*Escherichia coli* BL21 (DE3) cells were transformed with pBTR1-E104C plasmid to express *Homo sapiens* E104C hC*c* in a recombinant way. Protein expression was performed as described in ref.^11^. for WT hC*c*. The purification protocol of the E104C hC*c* mutant was previously described^35^. The soluble domain of *Arabidopsis thaliana* pC*c*_1_—containing a Cys residue at position 10 that facilitates the protein binding to an electrode—was expressed and purified similarly as in ref. ^13^, with minor modifications. *E. coli* BL21 (DE3) cells co-transformed with the plasmids pET_pC*c*_1_ and pEC86 were used to produce the soluble form of pC*c*_1_. Both E104C hC*c* and pC*c*_1_ samples were dialyzed against 10 mM sodium phosphate, pH between 6.5 and 7.0. Atomically flat Au(111) single-crystal disks of 10 mm diameter and 1 mm thickness (MaTecK) were flame annealed and electrochemically polished prior to use^28^. Au(111) electrodes were incubated with a 56.8 μM solution of pC*c*_1_. Apiezon^TM^ insulated gold (Au wire 0.25 mm in diameter; GoodFellow) ECSTM probes^28^ were incubated with a 12.5 μM solution of hC*c*. Protein solutions were used in sodium phosphate buffer 10 mM at pH=6.5. Incubations were performed at 4 °C overnight.

### ECSTM measurements

Distance-tunneling characteristics (I-z) were measured with a PicoSPM microscope head and a PicoStat bipotentiostat (Molecular Imaging) controlled by Dulcinea electronics (Nanotec Electrónica). This setup allows adjusting *U*_S_ and *U*_P_ independently. A homemade electrochemical cell was used in four-electrode configuration, using a 0.25 mm diameter Pt80/Ir20 wire (Advent) as a counter electrode and a miniaturized ultralow leakage membrane Ag/AgCl (SSC) reference electrode filled with 3 M KCl (World Precision Instruments). Electrode potentials are expressed against this reference. Prior to measurements, the electrochemical cell was cleaned with piranha solution (3:1 v/v solution of H_2_SO_4_ and H_2_O_2_). Caution: piranha solution is a strong oxidizer and a strong acid. It should be handled with extreme care, as it reacts violently with most organic materials. For protein experiments, the aqueous electrolyte solution was sodium phosphate buffer 50 mM (pH = 6.5). In the inhibition experiments, after recording the first set of I-z curves for pC*c*_1_–hC*c* interaction, the electrolyte was replaced by wild-type hC*c*^11^ 12.5 μM in the sodium phosphate buffer. Measurements in pure water (18 MΩ cm^−1^< 4 ppb TOC, Milli-Q, Millipore) were performed under potential control. All solvents were degassed with nitrogen and filtered through a 0.02 μm diameter sterile filter (Anotop) prior to use. The I-z curves were acquired by setting an initial current set point of 0.4 nA, turning the feedback loop off, and recording the probe current at 12 nm/s and constant bias. Retrace I-z curves were obtained along 15 nm of piezo retraction. A total of 150 curves (20 samples per point, 1024 points) were recorded for each independent experiment. Approach I-z curves were acquired by retracting the piezo 100 nm from the initial current set point and recording the probe current during the approach until saturation was reached (probe current = 10 nA). A total of 30 curves were acquired for each independent experiment (35 samples per point, 4096 points). At least three independent experiments were conducted in each case.

### Data processing

Data were acquired using WSxM 4.0 software, treated using a custom-generated MATLAB code (The MATHWORKS, Inc.; code available in the Supplementary Information), and analyzed with OriginPro 8.5.0 SR1 (OriginLab Corp.). Errors are indicated as standard deviation of the mean.

### Molecular dynamics simulations

The initial model of the hC*c* and pC*c*_1_ proteins was built from the PDB structure 3CX5^4^, using the Multiseq plugin of the VMD program^36,37^. The two proteins were separated along one axis by 3 nm. The MD simulation was set up employing the program LEaP included in the Amber suite of programs^38^ and the ff99SB protein force field. All titratable residues were modeled in their corresponding protonation state at pH 6.5, which was further checked by analysis of their intermolecular interactions. The heme parameters were taken from the AMBER parameters database (http://research.bmh.manchester.ac.uk/bryce/amber), considering RESP charges for the reduced and oxidized cases from Autenrieth et al.^39^. The system was solvated with explicit TIP3P water molecules^40^, and it was neutralized with one sodium atom, in addition to the 33 NaCl molecules to reproduce a concentration of 50mM. The final system contains 103610 atoms. Molecular dynamics simulations were performed using Amber14^38^. A restraint on the residues 1–15 (pC*c*_1_) and 97–104 (hC*c*) was applied to model the experimental conditions where Cys10 and Cys104 are attached to the gold probes. A thermal equilibration to 300 K was done prior to the equilibration dynamics in the NPT ensemble for 36 ns. The SHAKE algorithm, with an integration time step of 2 fs, was used. APBS electrostatics calculations^41^ were performed in the postprocessing stage to obtain the equipotential surfaces. The Volmap Plugin 1.1 was used to obtain average concentration maps. Figures were drawn with Gnuplot^42^ and VMD^36^.

## Electronic supplementary material

Supplementary Information

## Data Availability

Additional data that support the findings of this study are available from the corresponding author upon request.
