# Supplementary material for: Long distance electron transfer through the aqueous solution between redox partner proteins
Source: Nat Commun. 2018 Dec 4;9:5157. doi: 10.1038/s41467-018-07499-x (PMC6279779; doi:10.1038/s41467-018-07499-x)
Supplement: Supplementary file 1 — Supplementary Information [file 41467_2018_7499_MOESM1_ESM.pdf]

Supplementary Information

Long distance electron transfer through the aqueous solution between redox partner proteins

Anna Lagunas[1,2], Alejandra Guerra-Castellano[3], Alba Nin-Hill[4], Irene Díaz-Moreno[3], Miguel A. De la Rosa[3], Josep Samitier[1,2,5], Carme Rovira,[4,6] Pau Gorostiza.[1,2,6]

[1]Institute for Bioengineering of Catalonia (IBEC), The Barcelona Institute of Science and Technology (BIST), Barcelona 08028 Spain.
[2]Networking Biomedical Research Center (CIBER), Madrid 28029 Spain.
[3]Institute of Chemical Research (IIQ), Centre of Scientific Research *Isla de la Cartuja* (cicCartuja), University of Sevilla - CSIC, Sevilla 41092 Spain.
[4]Inorganic and Organic Chemistry Department & Institute of Theoretical and Computational Chemistry (IQTCUB), University of Barcelona (UB), Barcelona 08028 Spain.
[5]Department of Electronics and Biomedical Engineering: University of Barcelona (UB), Barcelona 08028 Spain.
[6]Catalan Institution for Research and Advanced Studies (ICREA), Barcelona 08010 Spain.

Correspondence and requests for materials should be addressed to Pau Gorostiza: pau@icrea.cat

Table of Contents

Supplementary Methods 3

Estimation of the extended protein length 3

Experimental details of the electrochemical gating and definition of the electrochemical gate potential 3

MATLAB code used in I-z data processing 3

Supplementary Figures 6

Supplementary Methods

Estimation of the extended protein length

The length of the extended proteins can be estimated from the corresponding amino acid (AA) sequences and assuming an averaged AA length of 0.375 nm:

hC*c* E104C

MGDVEKGKKI FIMKCSQCHT VEKGGKHKTG PNLHGLFGRK TGQAPGYSYT AANKNKGIIW GEDTLMEYLE NPKKYIPGTK MIFVGIKKKE ERADLIAYLK KATNC

pCc_1

ADE AEHGLECPNY PWPHEGILSS YDHASIRRGH QVYQQVCASC HSMSLISYRD LVGVAYTEEE AKAMAAEIEV VDGPNDEGEM FTRPGKLSDR LPEPYSNESA ARFANGGAYP PDLSLVTKAR HNGQNYVFAL LTGYRDPPAG ISIREGLHYN PYFPGGAIAM PKMLNDEAVE YEDGTPATEA QMGKDVVSFL SWAAEPEME

Experimental details of the electrochemical gating and definition of the electrochemical gate potential

I-z curves were recorded at different probe (U_P) and substrate (U_S) potentials, using a constant bias potential ($U_{bias} = U_P - U_S$). The potential of the reference electrode is adjusted to modulate the gate voltage in analogy to the gate electrode in a field effect transistor (FET). As the ECTS probe is grounded in our STM electronic configuration, the EC gate corresponds to $-U_S$ at any given bias.[29]

MATLAB code used in I-z data processing

```
clear all;
clc;

fileList = dir;

%Guess number of valid .txt files/max num of values in each file & reorder them
maxFileNum=0;
numOfTxtFiles=0;
maxNumOfValuesInEachTxtFile=0;
reorderedFileArray=cell(numel(fileList),1);
for index = 1:numel(fileList)
   curFileName=fileList(index).name;
   if strfind(curFileName,'.txt') > 0
       %Obtain file number and order it
```

```
      tempFind=strfind(curFileName,'_');
      fileNumber=curFileName(tempFind(end)+1:strfind(curFileName,'.txt')-1);
      reorderedFileArray{str2num(fileNumber)}=curFileName;

      %Obtain real number of .txt files
      currentFileValues=load(curFileName);
      if length(currentFileValues)>0 %Empty files must be avoided
         numOfTxtFiles=numOfTxtFiles+1;
         %Obtain max num of values
          if length(currentFileValues(:,2)) > maxNumOfValuesInEachTxtFile
            maxNumOfValuesInEachTxtFile = length(currentFileValues(:,2));
         end
      end

      %Obtain max file num
      if str2num(fileNumber) > maxFileNum
         maxFileNum = str2num(fileNumber);
      end
   end
end

%Obtain basefileName
curFileName=reorderedFileArray{1};
tempFind=strfind(curFileName,'_');
baseFileName=curFileName(1:tempFind(end)-1);

%File parsing
valuesMatrix=zeros(maxNumOfValuesInEachTxtFile,numOfTxtFiles*3);
processedFileNum=1;
for index=1:maxFileNum
   curFileName=reorderedFileArray{index};
   if length(curFileName) > 0 %Some file numbers may be missing
      currentFileValues=load(curFileName);
      fileLength=numel(currentFileValues)/2;
      if fileLength>0 %Empty files must be avoided
         %Find the minimum Y value
         minYvalue=min(currentFileValues(:,2));
         %Correct the read values
         correctedYvalues=currentFileValues(:,2)-min(currentFileValues(:,2));
         %Save the new file values within the matrix
         valuesMatrix(1:fileLength,processedFileNum)=currentFileValues(:,1);
         valuesMatrix(1:fileLength,processedFileNum+1)=currentFileValues(:,2);
         valuesMatrix(1:fileLength,processedFileNum+2)=correctedYvalues(:);
         processedFileNum=processedFileNum+3;
      end
   end
```

```
end

%Write the corrected values to an excel file
outputXcelFileName=['processed_' baseFileName '.xlsx'];
xlswrite(outputXcelFileName,valuesMatrix);
```

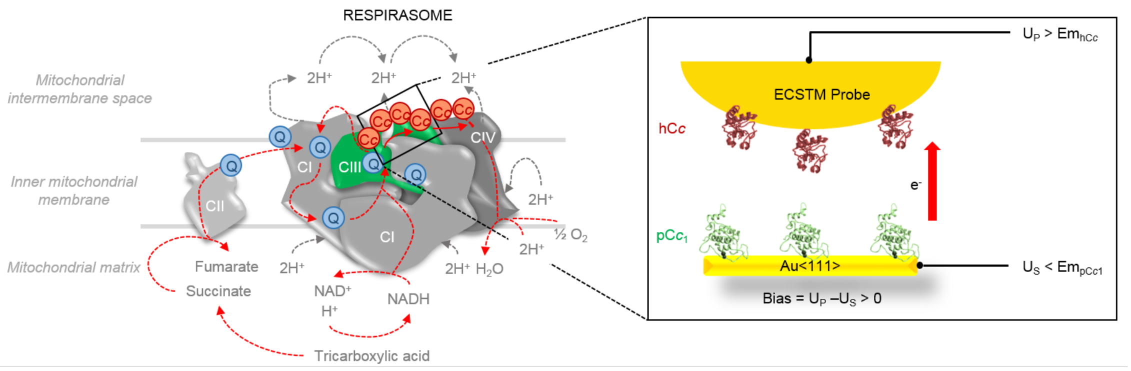

Supplementary Figure 1

The electrochemical potentials applied to the probe (hC*c*) and the sample (pC*c*$_1$) in the ECSTM set-up mimic the sense of the physiological electron flow. Schematic representation of the charge flow (depicted with arrows) through complex II (CII) and respirasome, which comprises complexes I (CI), III (CIII) and IV (CIV) in the mitochondrial respiratory chain. Red arrows indicate the sense of the electron flow from the ubiquinone (Q) pool, through CIII, cytochrome *c* (C*c*) and CIV to oxygen. The electron flow is associated with proton extrusion to the mitochondrial intermembrane space, which produces the electrochemical gradient used in ATP synthesis. ECTS experiments were performed at electrochemical potentials in which pC*c*$_1$ on the substrate is reduced ($U_S < Em_{pCc1}$) and hC*c* in the ECSTM probe is oxidized ($U_P > Em_{hCc}$), resulting in a constant positive bias. Under these conditions, electrons are transferred from pC*c*$_1$ to hC*c* in analogy to physiological ET.

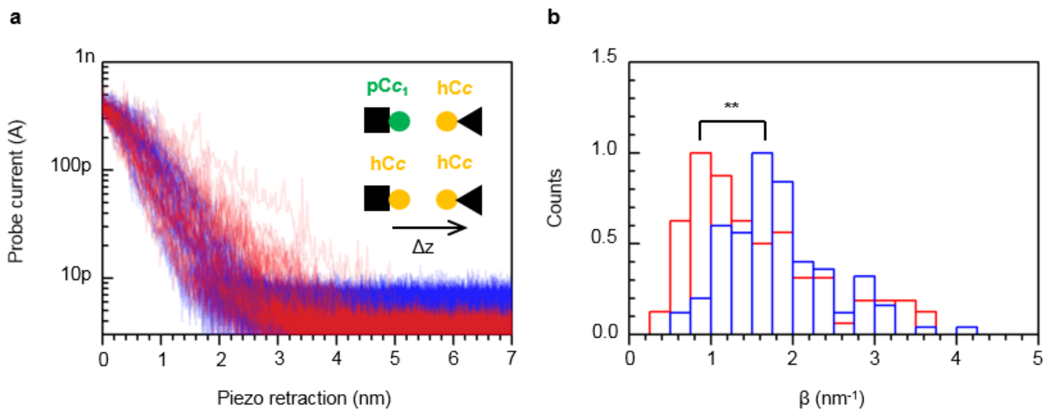

Supplementary Figure 2

Current-distance spectroscopy of specific ET between redox partners hC*c* (probe) and pC*c*$_1$ (sample), compared to the non-specific interaction between hC*c* in both probe and sample electrodes (self-ET). **a,** Ensemble of semi logarithmic retrace I-z plots showing that the ET current decay with the distance is more pronounced for hC*c*-hC*c* (self-ET; blue) than for pC*c*$_1$-hC*c* (red). **b,** Histograms of distance-decay constant β quantified from individual curves in **a**. Significant differences ($P < 0.01$; non-parametric Kolmogorov–Smirnov test) were found between pC*c*$_1$-hC*c* and hC*c*-hC*c* β distributions (red and blue histograms respectively), indicating that ECTS is sensitive to the specificity between the redox partner proteins (see also Fig.1cd and Suppl. Fig 3). Experiments were performed at U_S = -200 mV and U_P = 600 mV in 50 mM sodium phosphate buffer, pH = 6.5. Initial set point 0.4 nA.

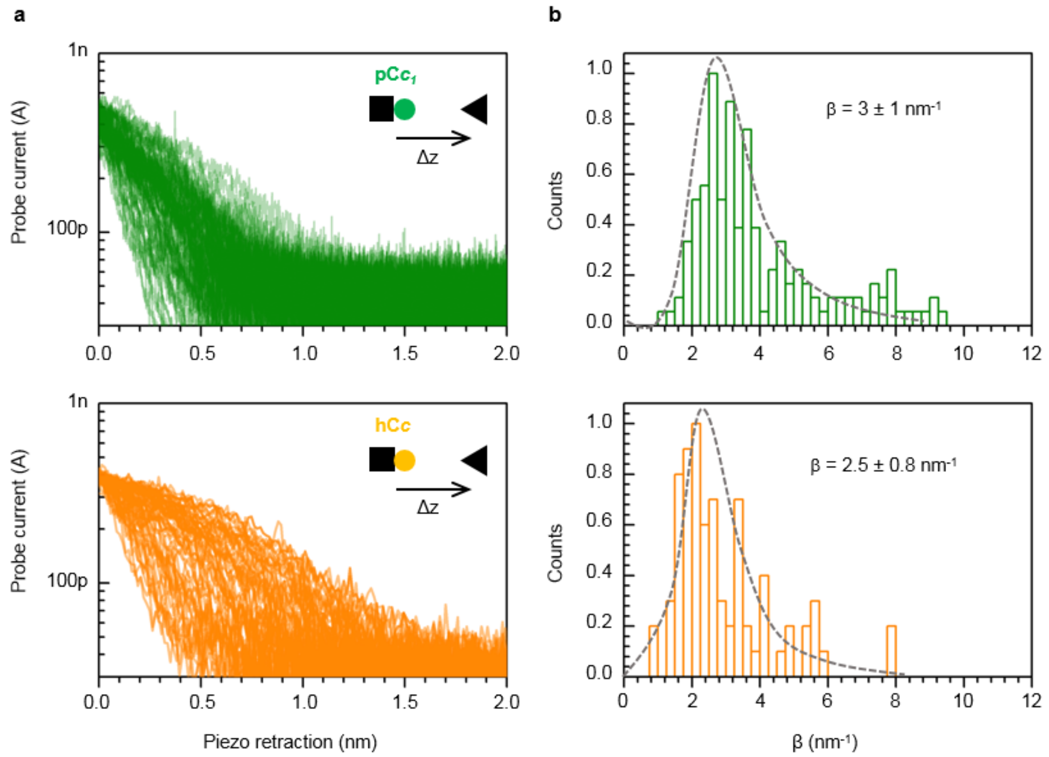

Supplementary Figure 3

Current-distance ECTS of the interaction of pC*c*1 or hC*c* with bare gold. **a,** Ensemble of semi logarithmic retrace I-z plots recorded for pCc_1 (green), and for hC*c*-coated gold (orange). **b,** Corresponding histograms of β quantified from individual curves in **a**. Dashed lines are an eye guide. β values (given as the mean with its standard error) are twice as high as the values obtained for the specific pCc_1-hC*c* interaction (Fig. 1), indicating that ECTS is sensitive to the presence of proteins in both electrodes. Experiments were performed at U_S = -200 mV and U_P = 600 mV in 50 mM sodium phosphate buffer, pH = 6.5. Initial set point 0.4 nA.

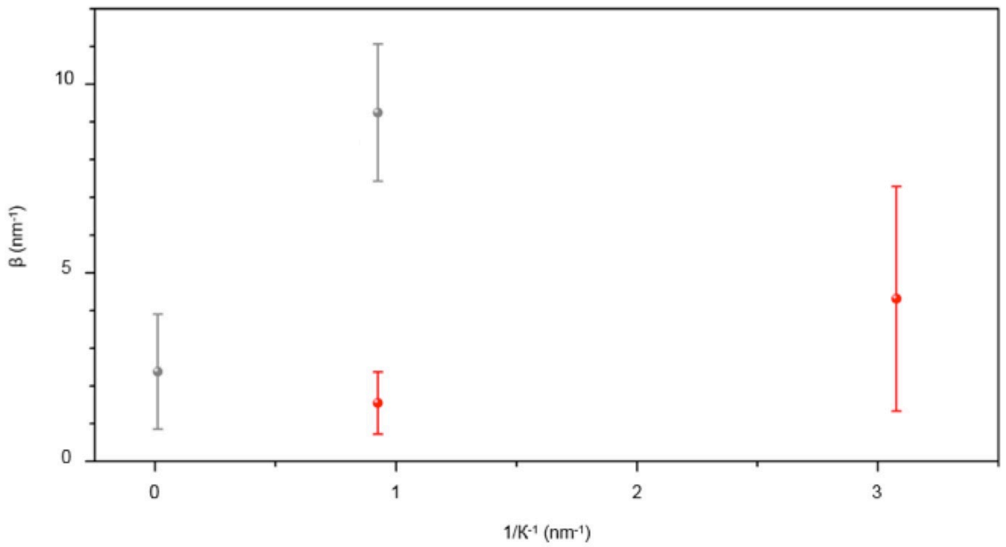

Supplementary Figure 4

Long distance ET between pCc_1-hC*c* is driven by the electrical double layer at the interface. The β values obtained experimentally for bare gold (grey) and for pCc_1-hC*c* (red) are correlated with the calculated inverse Debye-Hückel length (K^{-1}) at the same ionic concentration. It is observed that at low ionic concentration (low K^{-1}) the spatial range of ET is higher (the distance decay constant β is lower). The numerical correlation between β and $1/K^{-1}$ for pCc_1-hC*c* indicates that the current and potential between proteins are driven by similar distance-decay lengths.

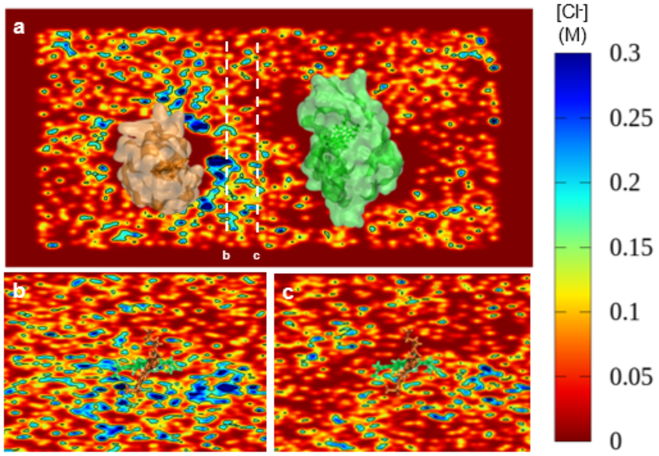

Supplementary Figure 5

Averaged chloride concentration maps from MD simulations. **a,** Side view of the averaged chloride concentration (M) map, showing that it is approximately homogenous between proteins except near the hC*c* (orange) surface due to binding to the lysine ring around the heme group (K13, K27, K72, K79, K86, K87). hC*c* and pCc_1 (green) proteins are superimposed for visualization purposes. Each contour line corresponds to 0.15 M. **b,c,** Cross sections corresponding to the dashed white lines in **a**. The two planes are separated 0.6 nm. The corresponding heme groups (hC*c* protein, orange and pCc_1 protein, green) are also placed on the figure for visualization purposes.

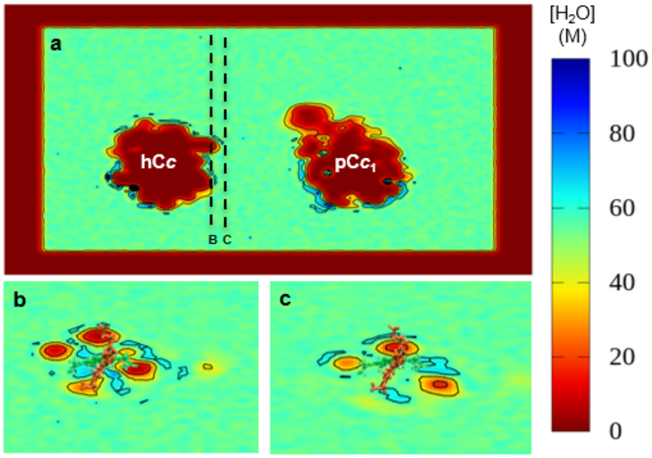

Supplementary Figure 6

Averaged water concentration maps from MD simulations. **a,** Side view of the averaged water concentration (M) map, showing that it is approximately homogenous between proteins except near the surface of hydrophilic residues such as Q16 and K13. Each contour line corresponds to 20 M. **b,c,** Cross sections corresponding to the dashed black lines in **a**. The two planes are separated 0.1 nm. The corresponding heme groups (hC*c* protein, orange and pCc_1 protein, green) are placed on the figure for visualization purposes.

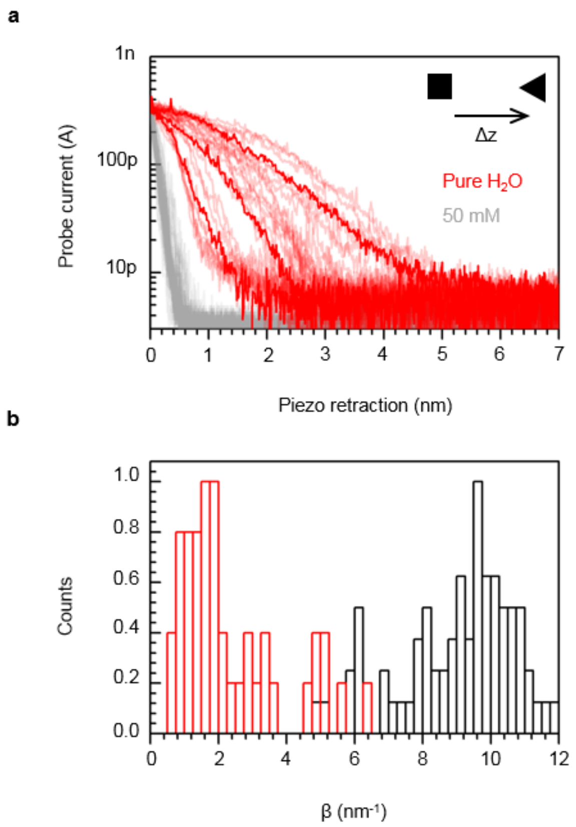

Supplementary Figure 7

Effect of the electrolyte in ECTS measurements in bare gold samples. **a,** Ensemble of semi-logarithmic probe retraction I-z plots obtained for bare gold in 50 mM sodium phosphate buffer, pH = 6.5 (grey) and in pure water (red), showing a more gradual current decay in the latter. Selected representative traces are depicted in bold. **b,** Histograms of β quantified from individual curves in **a**. All measurements were performed at a constant bias of 800 mV and at an initial set point of 0.4 nA.

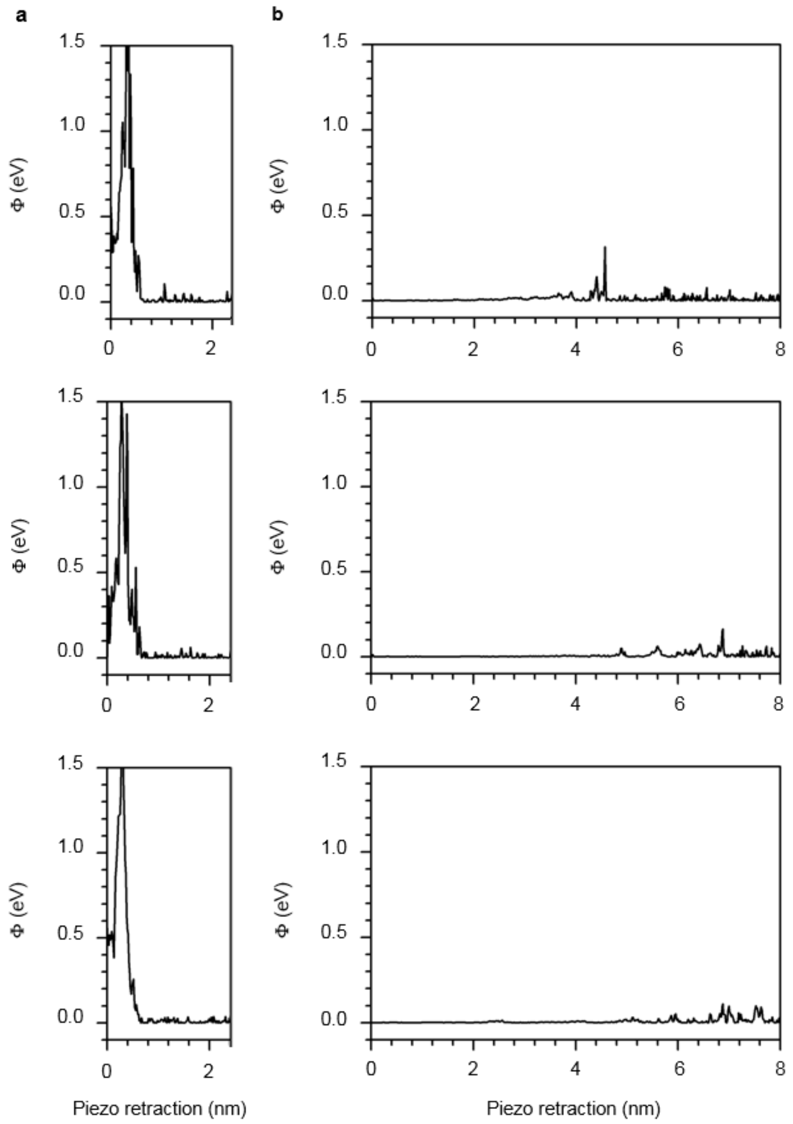

Supplementary Figure 8.

Activation-less electron conduction in pure water. Plot of the barrier height (Φ) dependence with distance calculated from experimental I-z curves following the procedure described in reference 27. **a,** In bare gold at 50 mM sodium phosphate buffer (pH = 6.5) the barrier oscillates between 0.5 and 1.2 eV with an average around $\Phi_{local,fit}$ = 0.8 ± 0.2 eV and a periodicity of 0.35 nm, in agreement with the results of reference 27. **b,** Calculated barrier-distance plots of bare gold in pure water yield Φ < 50 meV corresponding to activation-less conduction.

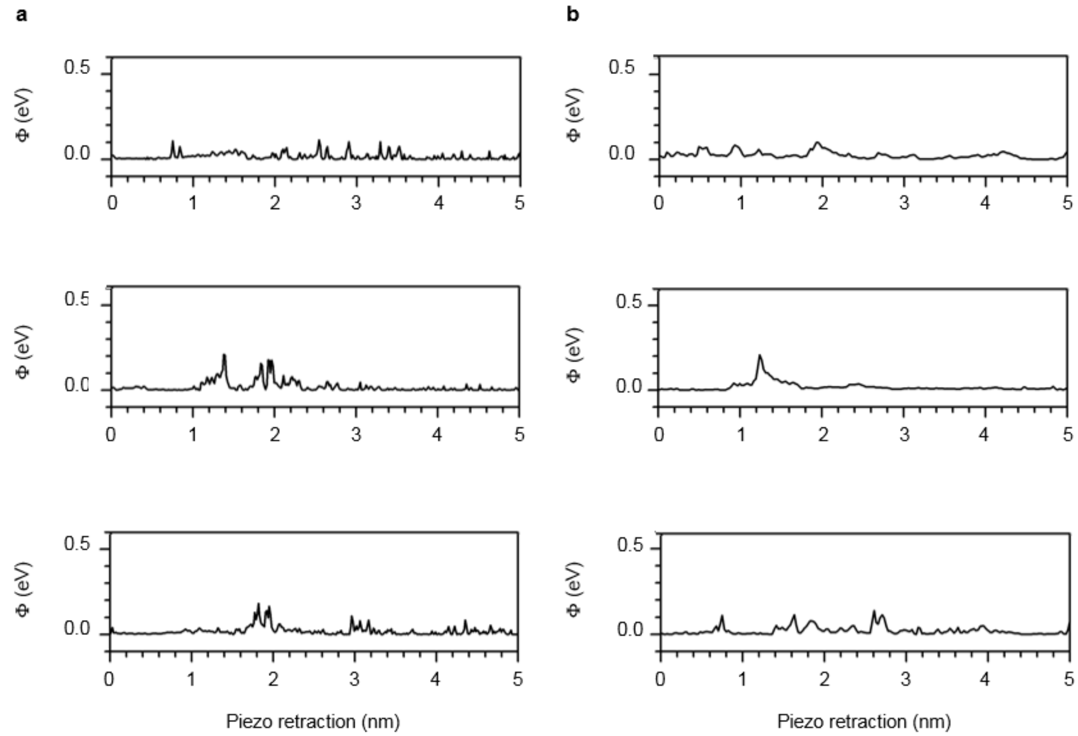

Supplementary Figure 9.

Activation-less electron conduction between pCc_1-hCc proteins. Plots of the calculated barrier height (Φ) as a function of the distance for pCc_1-hCc obtained from representative I-z curves of **a,** probe retraction experiments (Fig. 1A) and **b,** probe approach experiments after 100 nm retraction (Fig. 1E). The low barrier obtained in both cases (Φ < 50 meV) indicates activation-less conduction. Experiments were conducted in 50 mM sodium phosphate buffer, pH = 6.5.

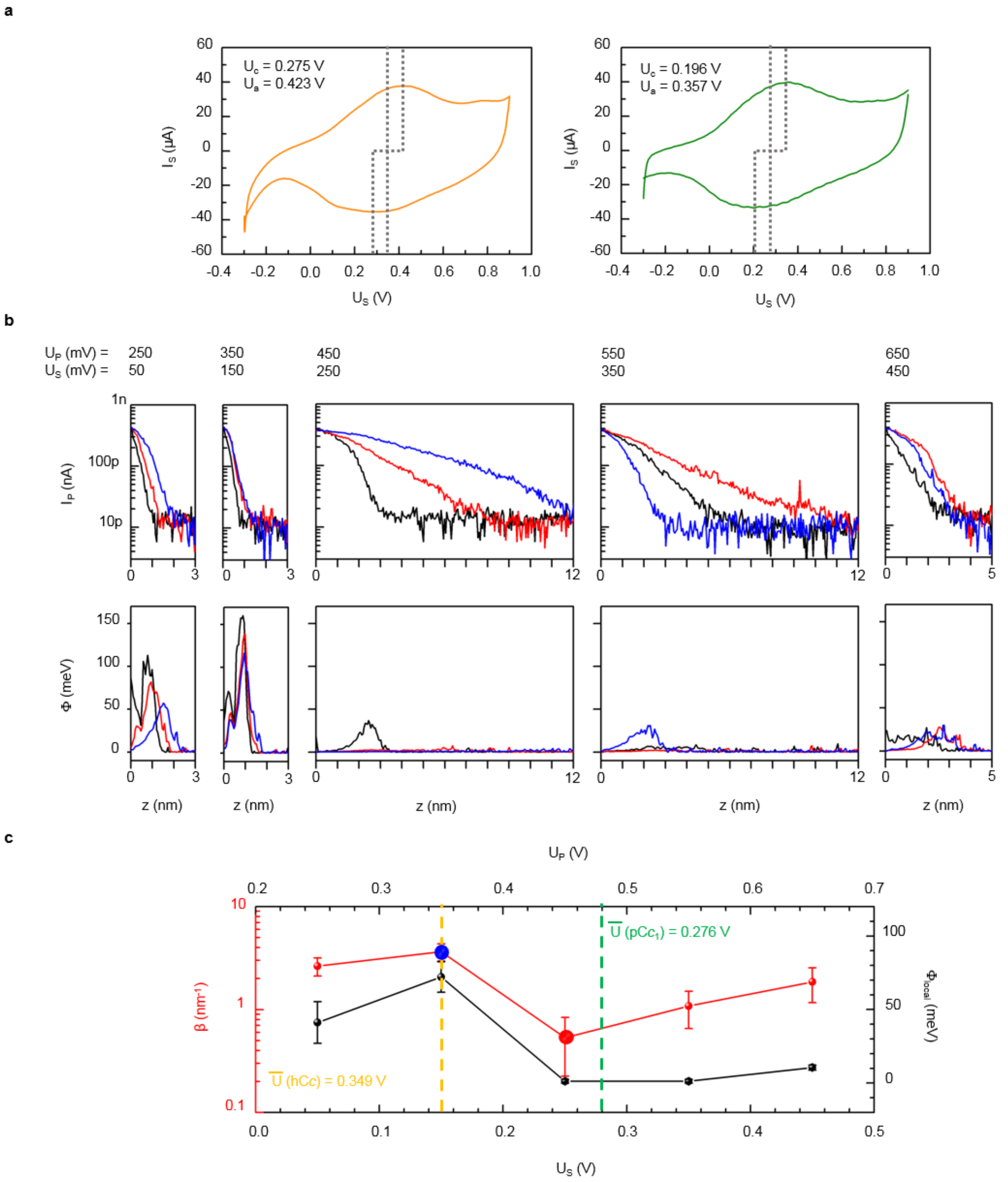

Supplementary Figure 10.

Distance decay factor (β) and local barrier height (Φ_{local}) reach minima near the redox potentials of p*Cc*$_1$-h*Cc* in electrochemically gated long distance ET. **a,** Cyclic voltammetry of Au(111) electrodes modified with h*Cc* (left) and p*Cc*$_1$ (right). The corresponding reduction (U_c) an oxidation (U_a) peaks for the Fe^{3+}/Fe^{2+} couple are indicated. **b,** Representative semi-logarithmic I-z plots obtained at 200 mV constant bias for p*Cc*$_1$-h*Cc* at the indicated probe (U_P) and sample (U_S) potentials (upper row) and the corresponding Φ-z plots calculated according to ref. 27 (lower row). Near U_S = 0.25 V/SSC the current decrease is more gradual and β and Φ_{local} are lower than at other

potentials. **c,** Plot of the averaged β (red) and Φ_{local} (black) values, obtained from individual I-z curves, as a function of sample (U_S) and probe (U_P) potentials. Minima are obtained near the redox midpoint potentials of hC*c* and pCc_1 (dashed lines).
